
JOURNAL INFORMATION
==============================
NLM Title Abbreviation: ScientificWorldJournal
Journal ID: ScientificWorldJournal
NLM Journal ID: 101131163
Journal ID: TSWJ

The Scientific World Journal

ISSN: 2356-6140
EISSN: 1537-744X
Publisher: Wiley

ARTICLE INFORMATION
==============================
PMCID: PMC5917397
PMID: 16604257
DOI: 10.1100/tsw.2006.83
Article ID: 379380
Article version: 1
Subjects: Directions in Science

Impairment of the Activity of Glycosaminoglycan-Binding Cytokines by Functionally Abnormal Heparan Sulfates: A Novel Mechanism Underlying Disease Pathophysiology

Gupta Pankaj *
Hematology/Oncology Section, Veterans Affairs Medical Center % Hematology-Oncology-Transplantation Division, Department of Medicine, University of Minnesota Medical School, Minneapolis, USA
*Pankaj Gupta: gupta013@umn.eduAcademic Editor: Martin Goette

Publication date: 2006
Electronic publication date: 2006 Apr 5
Volume: 6
First page: 452
Last page: 456
Received 2006 Mar 1; Accepted 2006 Mar 24
Copyright: Copyright © 2006 Pankaj Gupta.
Copyright year: 2006
License: This is an open access article distributed under the Creative Commons Attribution License, which permits unrestricted use, distribution, and reproduction in any medium, provided the original work is properly cited.
License URL: https://creativecommons.org/licenses/by/3.0/

Keywords: apoptosis, cytokines, fibroblast growth factor 2, glycosaminoglycans, heparan sulfate proteoglycans, heparitin sulfate, mucopolysaccharidosis-I, proteoglycans, receptors, cytokine, receptors, fibroblast growth factor, stem cells

Funding: U.S. Department of Veterans Affairs National Institutes of Health R03-HD-41411 National Institutes of Health R01-NS-48606 Children’s Cancer Research Fund University of Minnesota

==============================
Directions in Science
*TheScientificWorldJOURNAL* (2006) 6, 452–456
ISSN 1537-744X; DOI 10.1100/tsw.2006.83

### Impairment of the Activity of Glycosaminoglycan-Binding Cytokines by Functionally Abnormal Heparan Sulfates: A Novel Mechanism Underlying Disease Pathophysiology

Pankaj Gupta

*Hematology/Oncology Section, Veterans Affairs Medical Center & Hematology-Oncology-Transplantation Division, Department of Medicine, University of Minnesota Medical School, Minneapolis*

**E-mail**: gupta013@umn.edu

*Received March 1, 2006; Accepted March 24, 2006; Published April 5, 2006*

---

**KEYWORDS**: apoptosis; cytokines; fibroblast growth factor 2; glycosaminoglycans; heparan sulfate proteoglycans; heparitin sulfate; mucopolysaccharidosis-I; proteoglycans; receptors, cytokine; receptors, fibroblast growth factor; stem cells

---

In Hurler syndrome (mucopolysaccharidosis-I; MPS-I, the most common MPS disorder), an inborn deficiency of the lysosomal hydrolase α-L-iduronidase (IDUA) leads to incomplete degradation and lysosomal accumulation of heparan sulfate (HS) and dermatan sulfate (DS) glycosaminoglycans (GAGs; Fig. 1)[1]. The mechanisms by which accumulated GAGs cause multiorgan dysfunction, including the devastating neuropsychological manifestations of Hurler syndrome and other mucopolysaccharidoses, remain unclear. The neuropathology of Hurler syndrome includes neuronal loss leading to brain atrophy and formation of ectopic dendrites and synapses[2,3]. These features cannot be explained simply by the mechanical effect of GAG-distended lysosomes. It is well known that the fibroblast growth factor (FGF) cytokine family mediates cell growth and differentiation, as well as neurogenesis, axonal guidance, and neuronal survival[4,5,6]. The interaction of HS with FGF2 and its effect on the biological activity of FGF2 have been studied extensively[7]. In normal cells, structurally specific HS is required for optimal biological activity of FGF2[8], by formation of the "FGF2-FGF-receptor-HS" complex[9,10]. One structural feature required for facilitation of FGF2 activity is 6-O-sulfation of HS[11,12]. However, the consequences of structurally abnormal HS in a disease state on FGF2 activity and their contribution to disease pathophysiology have not been defined. We hypothesized that the functional properties of the abnormal-sized and sulfated HS in Hurler syndrome are defective, and that this leads to aberrant modulation of the biological activity of GAG-binding cytokines such as the FGFs, thereby contributing to the pathophysiology of Hurler syndrome (Fig. 2). Until recently, a major impediment to examining the developmental role of HS *in vitro* has been the lack of primary progenitor cells capable of differentiation into neuronal and other cell types representative of tissues affected by Hurler syndrome. This limitation has been removed by a major advance in cell biology: the isolation and culture of a multipotent, nonhematopoietic stem cell from human and rodent bone marrow (the multipotent adult progenitor cell: MAPC) capable of extensive expansion, and *in vitro* and *in vivo* differentiation into endodermal, mesodermal, and neuroectodermal cells representing all three embryonic lineages[13,14,15]. MAPCs,

©2006 with author.
Published by TheScientificWorld, Ltd.; www.thescientificworld.com

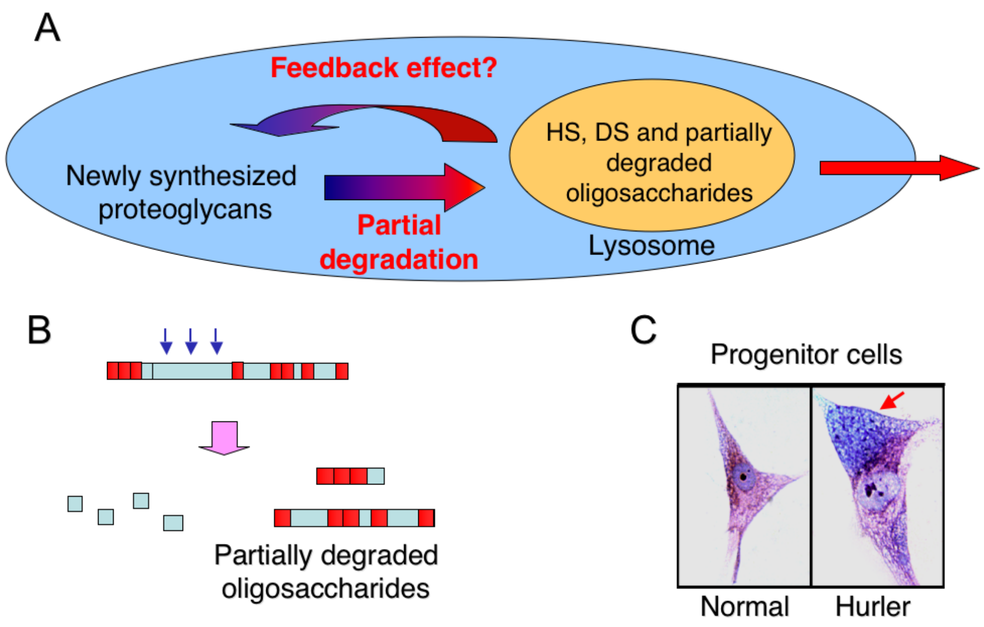

**FIGURE 1.** Lysosomal accumulation of partially degraded GAGs in Hurler syndrome. (A) Incompletely degraded HS and DS oligosaccharides accumulate in lysosomes. Some of these molecules are released outside the cell. The accumulated GAGs may possibly have feedback effects on the GAG biosynthetic pathways and on the expression of unrelated genes. (B) In the absence of IDUA, digestion of HS and DS chains into disaccharides is incomplete, since sequential digestion of the chains stops on reaching an iduronic acid residue (shown as red blocks). The remaining exoglycosidases and endoglycosidases are shown as blue arrows. (C) MAPC isolated from the bone marrow of patients with Hurler syndrome show lysosomal accumulation of GAGs, visible as a foamy blue region (red arrow).

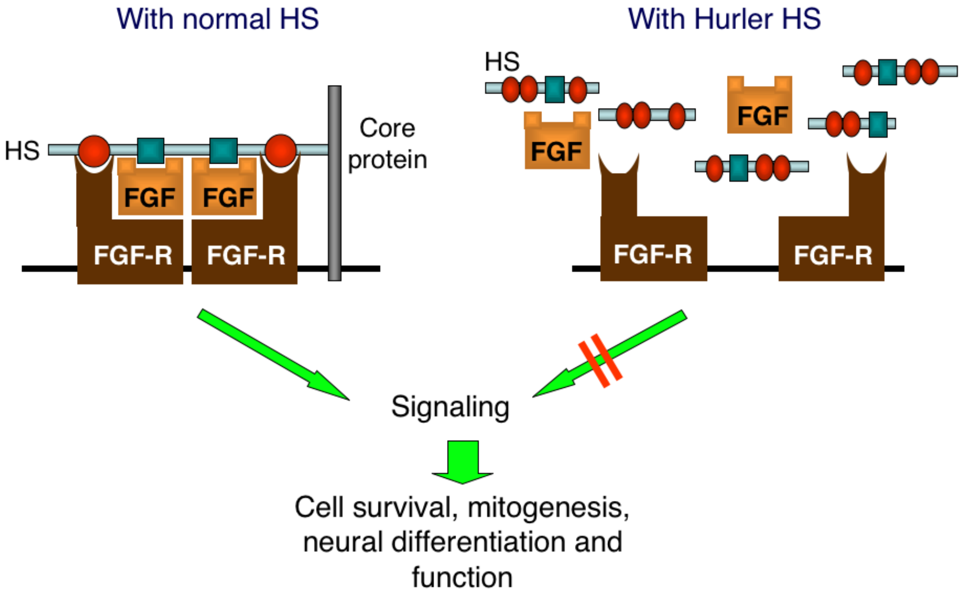

**FIGURE 2.** Hurler HS fails to mediate formation of the FGF2-FGFR1-HS complex. Normal HS proteoglycans on the cell surface mediate the formation and dimerization of the FGF2-FGFR1-HS complex, leading to the biological effects of FGF2. In contrast, the small, abnormally sulfated HS chains in Hurler syndrome fail to induce formation and dimerization of the FGF2-FGFR1-HS complex, impairing the biological activity of FGF2. The blue boxes and red circles on normal HS chains represent specific sulfated domains of HS required for binding to FGF2 and FGFR; abnormal Hurler HS domains (blue rectangles and red ovals) fail to bind.

therefore, provide an excellent *in vitro* model of development for studying the effect of HS on cytokine-mediated differentiation.

We used MAPC derived from normal donors and patients with Hurler syndrome to examine the structure and the functional properties of Hurler HS in our recent publication[16]. Using metabolic labeling of GAGs, we showed that the HS that accumulates in Hurler MAPC contained a large proportion of small ($M_r$ 5.5 kDa) polysaccharide chains. HPLC of fluorescently labeled disaccharides showed that the disaccharide composition of accumulated HS in Hurler MAPC was also markedly abnormal. While both normal and Hurler HS were comprised of the six major disaccharide types, the proportions of all three 6-O-sulfated disaccharides (UAGlcNAc6S, UAGlcNS6S, and UA2SGlcNS6S) were lower in Hurler HS. There was a progressive decline in the proportion of UAGlcNS6S (up to 84%) with increasing total accumulation of GAGs (correlation coefficient $R = 0.99$). This novel observation suggests that the structural aberration of HS in Hurler syndrome may continue to worsen with progression of the disease.

Our studies also demonstrated that the binding affinity of Hurler HS (containing lower proportions of 6-O-sulfated disaccharides) was reduced, which directly impaired the biological activity of FGF2. The binding of $^{125}$I-FGF2 to FGFR1 on Hurler MAPC was reduced by approximately 50%. Hurler HS also reduced $^{125}$I-FGF2 binding to normal MAPC. Removal of HS from Hurler MAPC and supplementation with normal HS restored $^{125}$I-FGF2 binding to Hurler MAPC. Thus, cell surface HS on Hurler cells directly interfered with $^{125}$I-FGF2 binding to FGFR1 and impaired formation of the FGF2-FGFR1-HS complex. Similarly, FGF2-induced proliferation of Hurler MAPC was lower than that of normal MAPC. This defect could also be corrected by removal of HS from Hurler MAPC and replacement by normal HS, indicating that Hurler HS interfered with the mitogenic signaling of FGF2. Further, Hurler HS also impaired the capability of FGF2 to promote cell survival, since about 40% of Hurler MAPC underwent apoptosis in serum-free, FGF2-supplemented medium, compared to about 20% normal MAPC ($p < 0.05$). Finally, the FGF2-induced proliferation of F32 cells (a HS-deficient, FGFR1-overexpressing cell line dependent on exogenously provided HS for FGF2 activity[10]) was lower in presence of Hurler HS than normal HS. The latter experiment confirmed that Hurler HS impaired the mitogenic signaling of FGF2. More recent, unpublished observations from our laboratory show that (a) Hurler HS also fails to induce ligand dimerization of FGF2, (b) the structural abnormalities of HS in Hurler MAPC are corrected rapidly by the presence of IDUA enzyme provided by normal cells, as is the defective neural differentiation of Hurler MAPC ("cross-correction"), and (c) the binding affinity of Hurler HS for various members of the FGF family, other cytokines, chemokines, and morphogens is also abnormal, suggesting that our observations with FGF2 are representative of a more generalized defect in modulation of cytokine function by HS.

Li et al.[17] recently reported that proliferation of neural progenitors (which depends on FGF2) is reduced in the subventricular zone of the brain of MPS-IIIB mice. Our *in vitro* observations are consistent with this *in vivo* phenomenon and together indicate that structurally abnormal HS hampers the mitogenic response of neural progenitors to GAG-binding cytokines. Patients with Hurler syndrome are clinically normal at birth, but develop progressive multisystem dysfunction starting in early childhood, with increasing tissue accumulation of GAGs. Our observation that increasing accumulation of total HS in Hurler MAPC is associated with progressive reduction in the UAGlcNS6S disaccharide leads to an interesting speculation: in the prenatal and early postnatal period, when tissue accumulation of GAGs is less, the structure of HS may not be abnormal enough to impair the biological activity of cytokines and morphogens required for embryogenesis and fetal development. However, as tissue GAG accumulation increases in infancy and early childhood, the structural abnormalities in HS worsen, leading to defective cytokine activity and the development of clinical features such as progressive mental retardation and other manifestations of neuronal dysfunction. Indeed, a recent study[18] showed that the higher levels of one specific HS and one specific DS trisaccharide in skin fibroblasts obtained from Hurler patients correlated with neuropathology. Importantly, this correlation was evident only after prolonged culture of the cells and progressive GAG accumulation.

The potential clinical implications of modulation of cytokine and chemokine function by HS proteoglycans are being increasingly appreciated[19]. Our studies may be indicative of a mechanism by which structurally and functionally abnormal HS directly contribute to disease pathophysiology and development, by perturbing the biological activity of critical cytokines and morphogens. These data also suggest that replacement of structurally abnormal HS by normal HS can restore cytokine signaling and biological response in neurodegenerative diseases where GAGs accumulate. The successful biochemical and functional cross-correction induced by normal MAPC also provide the rationale for therapeutic trials of normal or gene-corrected MAPC, which might serve as a source of missing enzyme(s) and directly differentiate into and replace lost neuronal and other cell types.

### ACKNOWLEDGMENTS

This work was supported by the U.S. Department of Veterans Affairs, NIH grants R03-HD-41411 and R01-NS-48606, the Children’s Cancer Research Fund, and the University of Minnesota Medical School.

### REFERENCES

1. Neufeld, E.F. and Muenzer, J. (2001) The mucopolysaccharidoses. In *The Metabolic and Molecular Basis of Inherited Disease.* Vol. 3. Scriver, C.R., Beaudet, A.L., Sly, W.S., Valle, D., Childs, B., Kinzler, K.W., and Vogelstein, B., Eds. McGraw-Hill, New York. pp. 3421–3452.
2. Walkley, S.U., Haskins, M.E., and Shull, R.M. (1988) Alterations in neuron morphology in mucopolysaccharidosis type I. A Golgi study. *Acta Neuropathol.* **75**, 611–620.
3. Purpura, D.P. and Suzuki, K. (1976) Distortion of neuronal geometry and formation of aberrant synapses in neuronal storage disease. *Brain Res.* **116**, 1–21.
4. Conrad, H.E. (1998) Fibroblast growth factors. In *Heparin Binding Proteins*. Academic Press, San Diego. pp. 301–349.
5. Vaccarino, F.M., Schwartz, M.L., Raballo, R., Rhee, J., and Lyn-Cook, R. (1999) Fibroblast growth factor signaling regulates growth and morphogenesis at multiple steps during brain development. *Curr. Top. Dev. Biol.* **46**, 179–200.
6. Alzheimer, C. and Werner, S. (2002) Fibroblast growth factors and neuroprotection. *Adv. Exp. Med. Biol.* **513**, 335–351.
7. Pye, D.A., Goodger, S.J., Hyde, P., and Gallagher, J.T. (2002) The regulation of the angiogenic growth factors FGF-1 and FGF-2 by heparan sulphate oligosaccharides is dependent on specific structural features. *TheScientificWorld, XXIst International Carbohydrate Symposium 2002*, 131–131.
8. Yayon, A., Klagsbrun, M., Esko, J.D., Leder, P., and Ornitz, D.M. (1991) Cell surface, heparin-like molecules are required for binding of basic fibroblast growth factor to its high affinity receptor. *Cell* **64**, 841–848.
9. Chang, Z., Meyer, K., Rapraeger, A.C., and Friedl, A. (2000) Differential ability of heparan sulfate proteoglycans to assemble the fibroblast growth factor receptor complex in situ. *FASEB J.* **14**, 137–144.
10. Krufka, A., Guimond, S., and Rapraeger, A.C. (1996) Two hierarchies of FGF-2 signaling in heparin: mitogenic stimulation and high-affinity binding/receptor transphosphorylation. *Biochemistry* **35**, 11131–11141.
11. Guimond, S., Maccarana, M., Olwin, B.B., Lindahl, U., and Rapraeger, A.C. (1993) Activating and inhibitory heparin sequences for FGF-2 (basic FGF). Distinct requirements for FGF-1, FGF-2, and FGF-4. *J. Biol. Chem.* **268**, 23906–23914.
12. Pye, D.A., Vives, R.R., Turnbull, J.E., Hyde, P., and Gallagher, J.T. (1998) Heparan sulfate oligosaccharides require 6-O-sulfation for promotion of basic fibroblast growth factor mitogenic activity. *J. Biol. Chem.* **273**, 22936–22942.
13. Jiang, Y., Henderson, D., Blackstad, M., Chen, A., Miller, R.F., and Verfaillie, C.M. (2003) Neuroectodermal differentiation from mouse multipotent adult progenitor cells. *Proc. Natl. Acad. Sci. U. S. A.* **100**, 11854–11860.
14. Jiang, Y., Jahagirdar, B.N., Reinhardt, R.L., Schwartz, R.E., Keene, C.D., Ortiz-Gonzalez, X.R., Reyes, M., Lenvik, T., Lund, T., Blackstad, M., Du, J., Aldrich, S., Lisberg, A., Low, W.C., Largaespada, D.A., and Verfaillie, C.M. (2002) Pluripotency of mesenchymal stem cells derived from adult marrow. *Nature* **418**, 41–49.
15. Reyes, M., Lund, T., Lenvik, T., Aguiar, D., Koodie, L., and Verfaillie, C.M. (2001) Purification and ex vivo expansion of postnatal human marrow mesodermal progenitor cells. *Blood* **98**, 2615–2625.
16. Pan, C., Nelson, M.S., Reyes, M., Koodie, L., Brazil, J.J., Stephenson, E.J., Zhao, R.C., Peters, C., Selleck, S.B., Stringer, S.E., and Gupta, P. (2005) Functional abnormalities of heparan sulfate in mucopolysaccharidosis-I are associated with defective biological activity of FGF-2 on human multipotent progenitor cells. *Blood* **106**, 1956–1964.
17. Li, H.H., Zhao, H.Z., Neufeld, E.F., Cai, Y., and Gomez-Pinilla, F. (2002) Attenuated plasticity in neurons and astrocytes in the mouse model of Sanfilippo syndrome type B. *J. Neurosci. Res.* **69**, 30–38.
18. Fuller, M., Brooks, D.A., Evangelista, M., Hein, L.K., Hopwood, J.J., and Meikle, P.J. (2005) Prediction of neuropathology in mucopolysaccharidosis I patients. *Mol. Genet. Metab.* **84**, 18–24.

19. Gotte, M. and Echtermeyer, F. (2003) Syndecan-1 as a regulator of chemokine function. *TheScientificWorldJOURNAL* **3**, 1327–1331.

**This article should be cited as follows:**

Gupta, P. (2006) Impairment of the activity of glycosaminoglycan-binding cytokines by functionally abnormal heparan sulfates: a novel mechanism underlying disease pathophysiology. *TheScientificWorldJOURNAL* **6**, 452–456. DOI 10.1100/tsw.2006.83.

### BIOSKETCH

**Pankaj Gupta** obtained his medical degree in 1983 from the All India Institute of Medical Sciences, New Delhi, followed by a residency in Internal Medicine (1984–1987) and subspecialty training in Medical Oncology (1987–1990) from the same institution. In 1991, he was awarded the Commonwealth Scholarship and the PN Berry Scholarship for further training and research in bone marrow transplantation at the Royal Marsden Hospital in the U.K. Working in the laboratory of Dr. Catherine Verfaillie during his fellowship at the University of Minnesota, Minneapolis (1991–1994), Dr. Gupta identified a stromal heparan sulfate that supported the long-term *in vitro* maintenance of human hematopoietic stem cells via modulation of cytokine and chemokine activity. Since February 1995, he has been on the faculty at the University of Minnesota Medical School (currently, Associate Professor of Medicine) and a staff hematologist-oncologist at the Minneapolis Veterans Affairs Medical Center. His current research interests include (a) examining the role of heparan sulfate proteoglycans in human hematopoiesis, particularly their interactions with cytokines and effect on primitive hematopoietic stem cells; (b) defining the pathophysiological mechanisms responsible for the clinical manifestations of mucopolysaccharidosis-I, and the effect of intracerebroventricular transplantation of multipotent progenitor cells in a murine model of MPS-I; and (c) studying the molecular basis for the myelodysplastic syndromes and conducting clinical trials of novel biological therapies in these diseases.
